# Unequal causes and consequences of AI use

**DOI:** 10.1038/s44271-026-00551-3

**Published:** 2026-11-11

**Authors:** Cydney H. Dupree, Yinghui Liang

**Affiliations:** School of Management, University College London, London, UK

**Keywords:** Human behaviour, Business and management

## Abstract

Generative AI enters organizations rife with inequality. Here, we propose a recursive process whereby inequality can drive marginalized group members to use AI, but actual or suspected AI use can reinforce inequality through unequal judgement of communicators’ capability, credibility, and authenticity.

Generative artificial intelligence is one of the most consequential technological advances of our time, but this technology does not enter into a vacuum. It enters a world pervaded with inequalities that people must navigate every day in diverse, unequal organizations. We propose a recursive process that links the use of generative AI to structural inequality, social cognition, and intergroup interactions—leading to a vicious cycle of AI use, stereotyping, and discrimination (see Fig. 1).

**Fig. 1:**
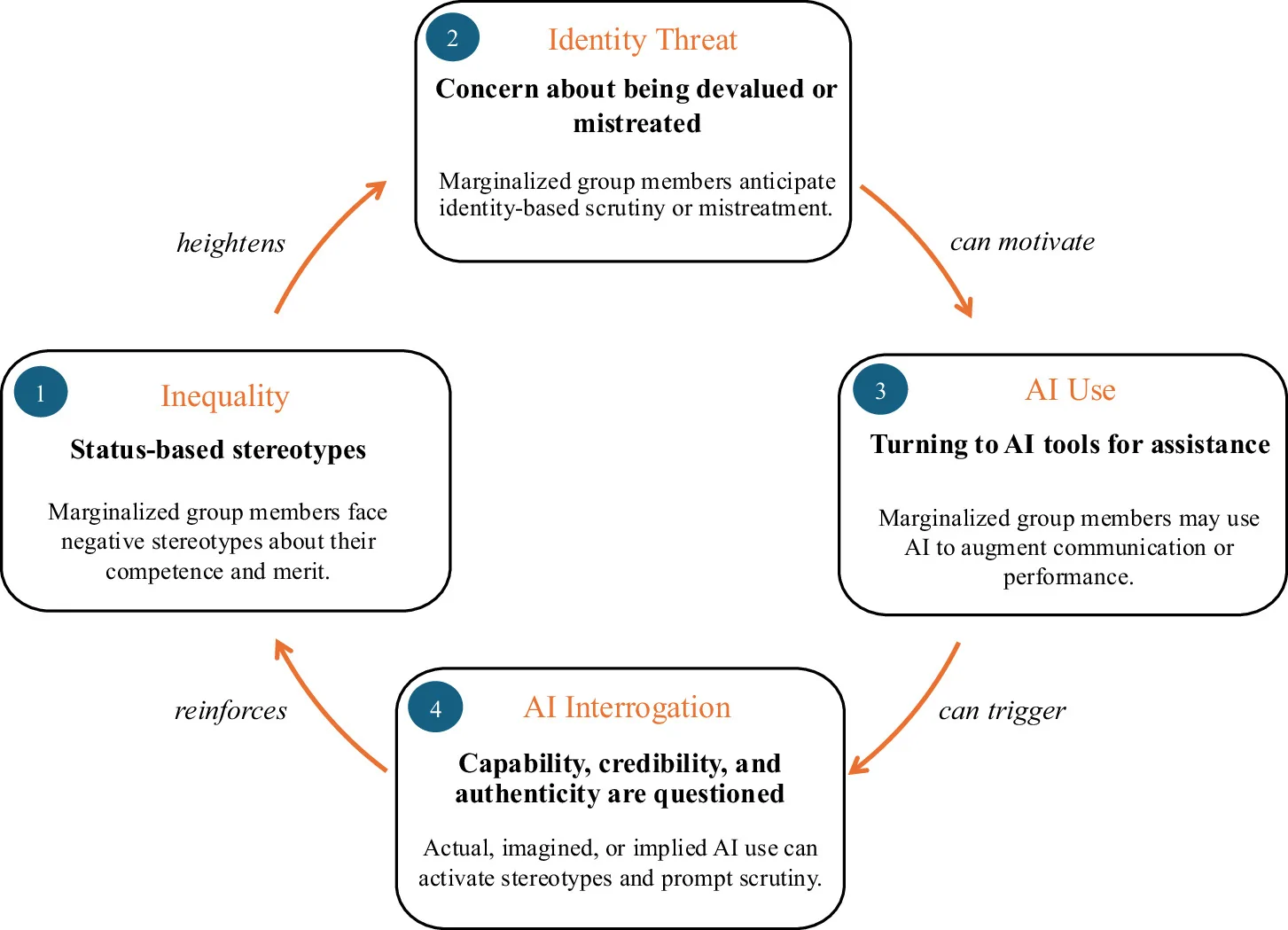
The recursive cycle of inequality and AI use. Existing inequalities and concurrent status-based stereotypes can heighten social identity threat among marginalized group members, which can motivate some to use generative AI to augment their communication or performance. However, actual, imagined, or implied AI involvement can prompt AI interrogation—questioning of communicators’ capability, credibility, and authenticity—which can reinforce existing inequality. Arrows represent hypothesized relationships.

## A recursive cycle: from inequality to AI use and back

### Social identity threat drives AI use

Consider the workplace. In racialized organizations, Black and Brown employees are more scrutinized than white employees. In male-normed offices, women are labeled as less suitable for intellectually demanding tasks. In neurotypical and ableist workplaces, neurodivergent and disabled workers face discrimination rooted in assumed incompetence. Within diverse, unequal organizations, marginalized group members often experience social identity threat: the fear of being devalued or mistreated due to one’s social identity^1^. As a result, they become anxious, closely monitor how others react to them, and may perform worse during exams, interviews, and presentations.

People may use AI tools for multiple reasons, including organizational policies that encourage use, a desire to increase productivity, or curiosity as AI agents become more sophisticated and use becomes more normative. However, marginalized group members may turn to AI—a readily available, anonymous, (seemingly) unbiased collaborator—as a means to reduce social identity threat. There is precedent for marginalized group members using technological tools to reduce threat, with positive effect. For instance, women who hide their social identities through avatars experience less stereotype threat and performance penalties^2^. Using generative AI may mitigate social identity threat by helping with research, double-checking drafts, or ensuring emails meet professional standards. Regardless of the reason for AI use, how its use is judged will vary. We propose that, while marginalized group members may reach for this tool to reduce the risk of being devalued or mistreated, using AI may ironically increase such risk.

### AI use activates stereotypes

For centuries, humans have evaluated other people based on how they present themselves: in person, in print, or online. Generative AI disrupts this fundamental process of inference from communication to communicator. The technological communication that powers knowledge economies and social media platforms can now be produced by a human, an artificial system, or both. This introduces a new attributional ambiguity: Is a person’s technologically-mediated communication uniquely their own, or has it been generated or shaped by AI?

Moral judgments of AI use will depend on who is using it. Students and office workers could use AI similarly, but the student may be seen as cheating while the worker may be seen as being efficient. Stereotypes play a role in these differential interpretations. Stereotypes are widely shared, subconsciously activated, and automatically applied, and they tend to depict lower-status group members as lacking in competence and motivation^3^. In contrast, AI and its users tend to be depicted as socially advantaged—prestigious, wealthy, white, masculine, and young^4^. It follows, then, that for advantaged group members, using AI is more likely to be seen as normative (and harmless). However, we contend that, for marginalized group members, using these tools is more likely to be seen as a way to cover for stereotypical attributes of incompetence and lack of motivation. Thus, the Black student who may or may not have used AI would be quickly dismissed as lazy, the woman employee discounted as dependent, and the neurodivergent job candidate rejected as incapable.

### Stereotypes incite AI interrogation

**Fig. 2:**
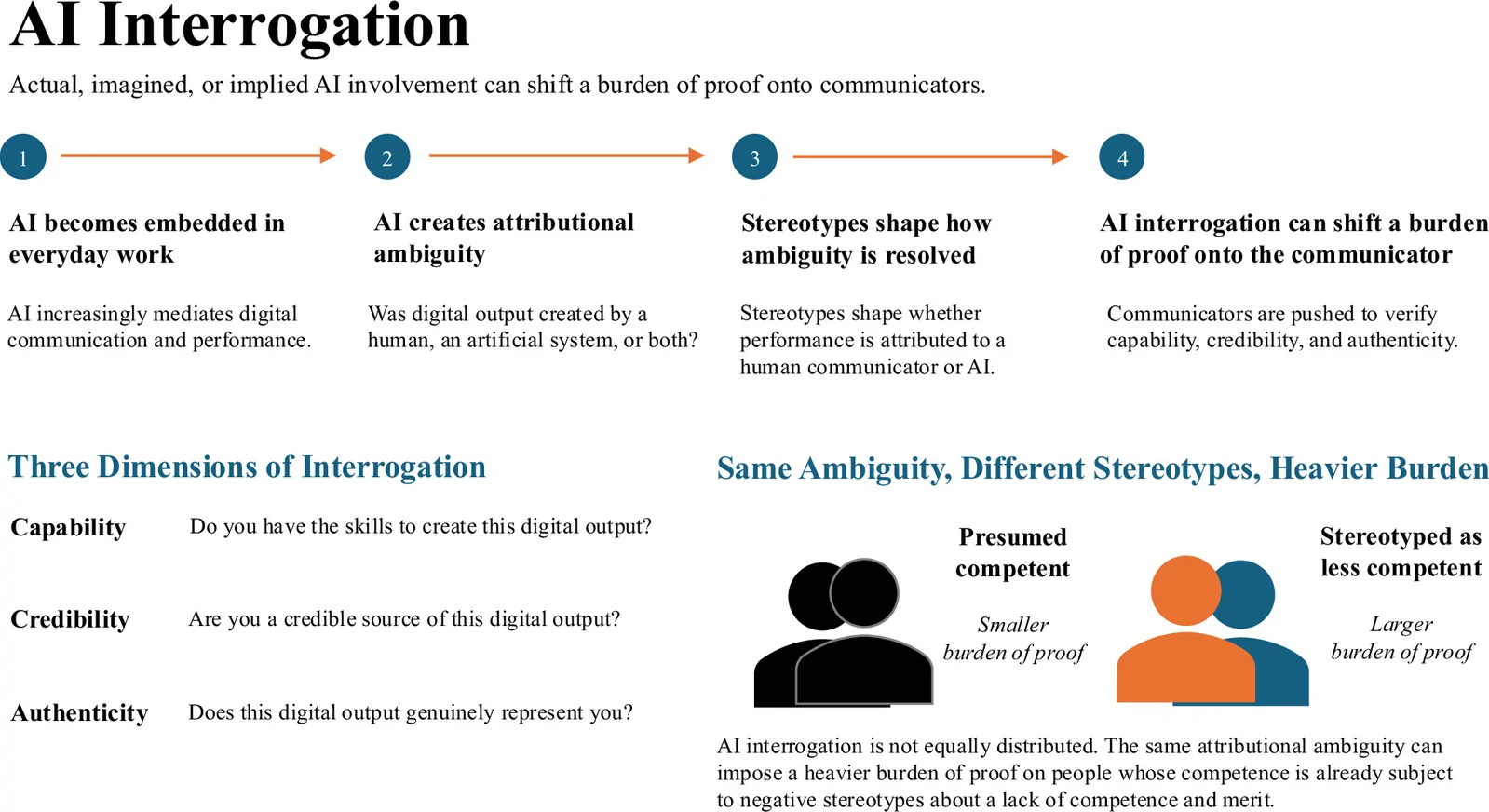
The process and unequal burden of AI interrogation. As AI becomes embedded in everyday work, AI creates attributional ambiguity about whether digital communication reflects a human, an artificial system, or both. Stereotypes can shape how ambiguity is resolved, shifting a burden of proof onto communicators to verify their capability, credibility, and authenticity. Capability concerns whether a communicator possesses the skills reflected in digital output; credibility concerns whether the communicator can be trusted as a reliable source of digital output; and authenticity concerns whether the digital output genuinely represents the communicator’s voice. The ambiguity created by increasingly AI-powered workplaces, schools, and social media may impose a higher burden of proof on people whose competence is already negatively stereotyped and whose merit is already questioned.

Stereotypes don’t just live in the mind; they shape everyday interactions with others^5^. In this case, we posit that stereotype activation can result in AI interrogation—questioning about one’s capability, credibility, and authenticity due to the actual, imagined, or implied involvement of generative AI. Marginalized group members will likely be most vulnerable to such scrutiny. In academia, scholars of color uniquely experience objectivity interrogation: written and verbal questioning about their scientific rigor in academic settings that arises from positivist norms^6^. In the classroom, evidence suggests that Black students are more than twice as likely as white students to be falsely accused of AI plagiarism^7^. As AI becomes ever present in schools and workplaces, all may experience such AI interrogation, but marginalized group members will be especially vulnerable to this unique form of discrimination (see Fig. 2).

## How to manage AI interrogation

How will people navigate AI interrogation? Three paths emerge.

### Decline AI use

Some will decline using AI. This may not be a bad thing. Though AI is being pushed relentlessly onto the public by giant, well-funded technology companies, it inflicts harm on people and the planet. Its models are trained on creative workers’ output without consent, credit, or compensation, while its infrastructure magnifies greenhouse gas emissions, pressures electrical and water supplies, and exacerbates long-standing inequalities by demanding land, electricity, and water of vulnerable communities. Some will decline AI use due to ethical, environmental, or social costs—and a belief that doing so will save them from AI interrogation. However, they may miss out on efficiency benefits. While the evidence of AI’s effect on actual productivity is decidedly mixed^8^, those who decline using AI must contend with the possibility of disadvantage.

### Document AI use

Others will use AI but strictly document use. Increasingly, students keep detailed assignment records in case of AI interrogation, and researchers submit AI disclosure statements to journals and funding agencies. In workplaces, marginalized workers may be more inclined (or more often asked) to document their AI use than advantaged workers, costing them valuable time and effort. Moreover, as noted, explicitly disclosing the use of AI may in fact increase the very stereotypes and interrogation they seek to avoid.

### Disguise AI use

A final group will use AI but disguise it. Marginalized group members are already more likely to cover stigmatized identities at work^9^, such as LGBTQ employees passing as straight, racially marginalized employees codeswitching, women hiding pregnancy and menopause, or neurodivergent employees suppressing natural traits. A survey of over 48,000 employees worldwide found that 57% disguise AI^10^. One reason may be threat: employees may disguise AI use to avoid being seen as less competent or replaceable. But masking is exhausting, and marginalized group members are already more likely to take on this burden to avoid stigma. AI use will be no exception.

## How to disrupt AI interrogation

In modern workplaces, the use of AI can feel empowering, reducing threat among those who face negative stereotypes. Simultaneously, the risk of AI interrogation can result in discrimination, leaving marginalized group members feeling patronized, excluded, and exhausted. Below, we offer recommendations to help reduce AI interrogation and its harm.

### Policymakers

Policymakers within and outside schools and workplaces must implement strong policies on AI use and disclosure requirements. Leaders must communicate those policies clearly and enforce them equally. Organizations should examine demands for proof of authorship, shifting the burden off the employees by building AI use documentation into workflows rather than requiring students and employees to supply evidence on their own. Regular audits can ensure that any demands are being applied consistently—and not selectively on those whose competence and belonging are already more likely to be questioned. The onus is ultimately on leaders to set clear policies and boundaries around AI use.

### AI users

As AI capabilities increase, we should all carefully consider whether, when, and how to use AI—and the ethical implications. Such judgment is itself becoming a form of competence and agency in modern schools, workplaces, and social media platforms. For those navigating unequal schools and workplaces, the growing presence of AI means that the burden of proof will increasingly fall on those already subjected to negative stereotypes. All—but especially members of marginalized groups—should consider keeping a record of their AI use, such as chat transcripts and version histories. Such records may prove useful in instances of interrogation.

### Scholars

In this great race to study AI, scholars must study AI through the lens of the racialized, gendered, and classed organizations in which these tools will be used. Research can contribute to our understanding of how existing inequalities shape who uses AI—and how its use may, in turn, reify inequalities. This research should be functional, cross-level, and embedded in people’s lived experiences.

As workplaces, schools, and social media platforms remain diverse but unequal, social status may determine who reaches for the assistance of generative AI out of desire versus necessity—and whether such assistance is accepted as harmless or denigrated as evidencing incompetence. To fully understand AI’s impact, we must examine how its everyday use shapes and is shaped by our social world. Only then can we maximize the promises and minimize the pitfalls of this increasingly powerful and ubiquitous digital tool.

## Supplementary information

Peer Review File

## Data Availability

This research did not generate any data.
