# Supplementary material for: Unequal causes and consequences of AI use
Source: Commun Psychol. 2026 Nov 11;4:161. doi: 10.1038/s44271-026-00551-3 (PMC13754788; doi:10.1038/s44271-026-00551-3)
Supplement: Supplementary file 1 [file 44271_2026_551_MOESM1_ESM.pdf]

nature portfolio

Peer Review File

Unequal Causes and Consequences of AI Use

Corresponding Author: Dr Cydney Dupree

This file contains all reviewer reports in order by version, followed by all author rebuttals in order by version.

Open Access This Peer Review File is licensed under a Creative Commons Attribution 4.0 International License, which permits use, sharing, adaptation, distribution and reproduction in any medium or format, as long as you give appropriate credit to the original author(s) and the source, provide a link to the Creative Commons license, and indicate if changes were made.
In cases where reviewers are anonymous, credit should be given to 'Anonymous Referee' and the source.
The images or other third party material in this Peer Review File are included in the article's Creative Commons license, unless indicated otherwise in a credit line to the material. If material is not included in the article's Creative Commons license and your intended use is not permitted by statutory regulation or exceeds the permitted use, you will need to obtain permission directly from the copyright holder.
To view a copy of this license, visit https://creativecommons.org/licenses/by/4.0/

UCL School of Management
University College London
One Canada Square, London E14 5AA, UK

September 24, 2026

Re: *Unequal Causes and Consequences of AI Use (COMMSPSYCHOL-26-0833)*

Dear Dr. Jennifer Bellingtier,

We were pleased to receive comments on our submission, now titled *Unequal Causes and Consequences of AI Use*, which was described as "interesting" and "easily understood". We have carefully responded to all editorial team comments. Below is a point-by-point clarification.

1. The **Editorial Team (E)** suggested that we revise our references to "interpretations of AI use" to the more specific and accurate "(moral) judgements of AI use".

REPSONSE: We appreciate this suggestion. We agree that our descriptions more closely capture moral judgements and have revised phrasing accordingly.

2. **E** also noted one area that could be cut for redundancy (p. 3, para. 1) and one area that could be reworded for clarity (p. 4, para. 3).

RESPONSE: We agree and have incorporated the suggested changes for clarity and concision.

3. **E** made several helpful suggestions regarding removal and revision of references and revision of highly specific references.

RESPONSE: We are grateful to the editorial team for these suggestions, which are appropriate for a Comment. We have removed references as suggested and revised references to Reviews where possible.

4. **E** noted that the notion of AI interrogation is well-described within the manuscript, but they suggested that this concept does not belong in the preface, as it is more easily understood as a process than a label.

RESPONSE: We agree that removal of this label in the preface will maximize comprehension. We have thus removed AI interrogation from the preface and revised accordingly. The preface now reads:

Generative AI enters organizations rife with inequality. Here, we propose a recursive process whereby inequality can drive marginalized group members to use AI, but actual or suspected AI use can reinforce inequality through unequal judgement of communicators' capability, credibility, and authenticity.

In closing, we hope you will agree that the manuscript has addressed the editorial team's concerns thoroughly and concisely. We appreciate the opportunity to resubmit this revised Comment to *Communications Psychology*. Thank you for your consideration.

Sincerely,
The Authors
